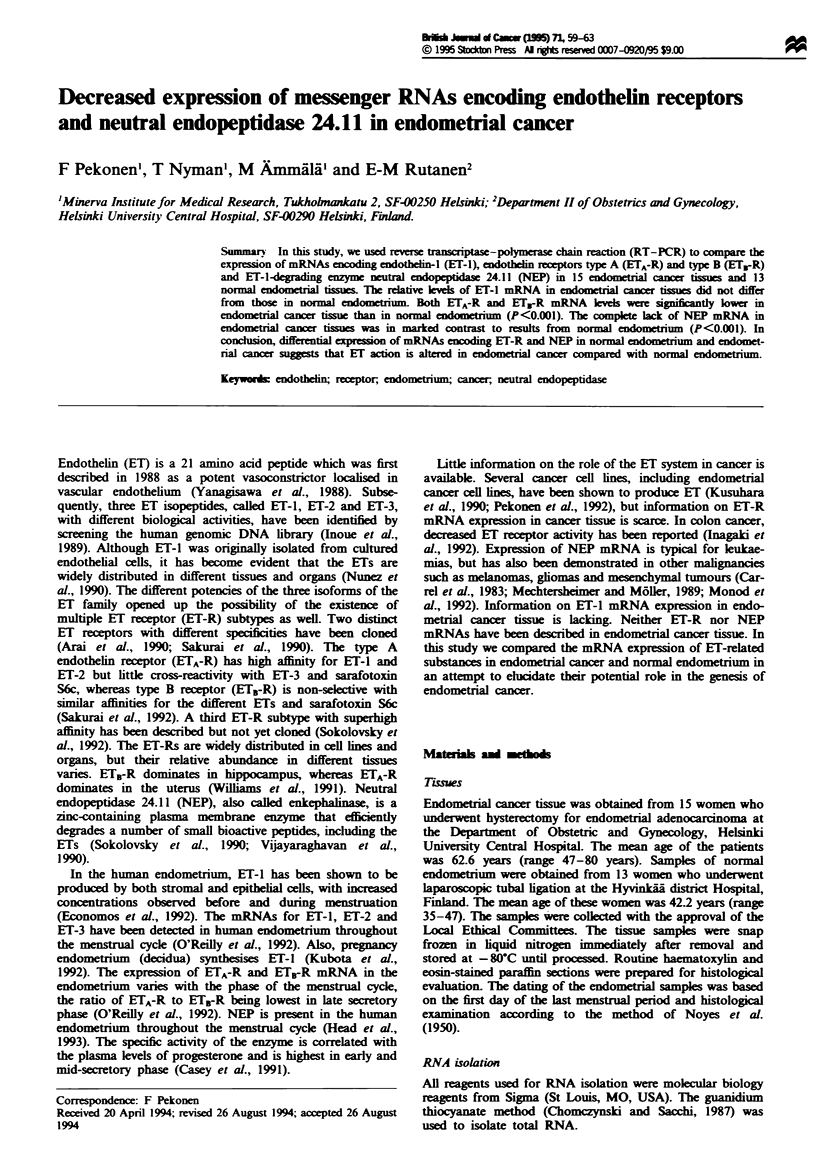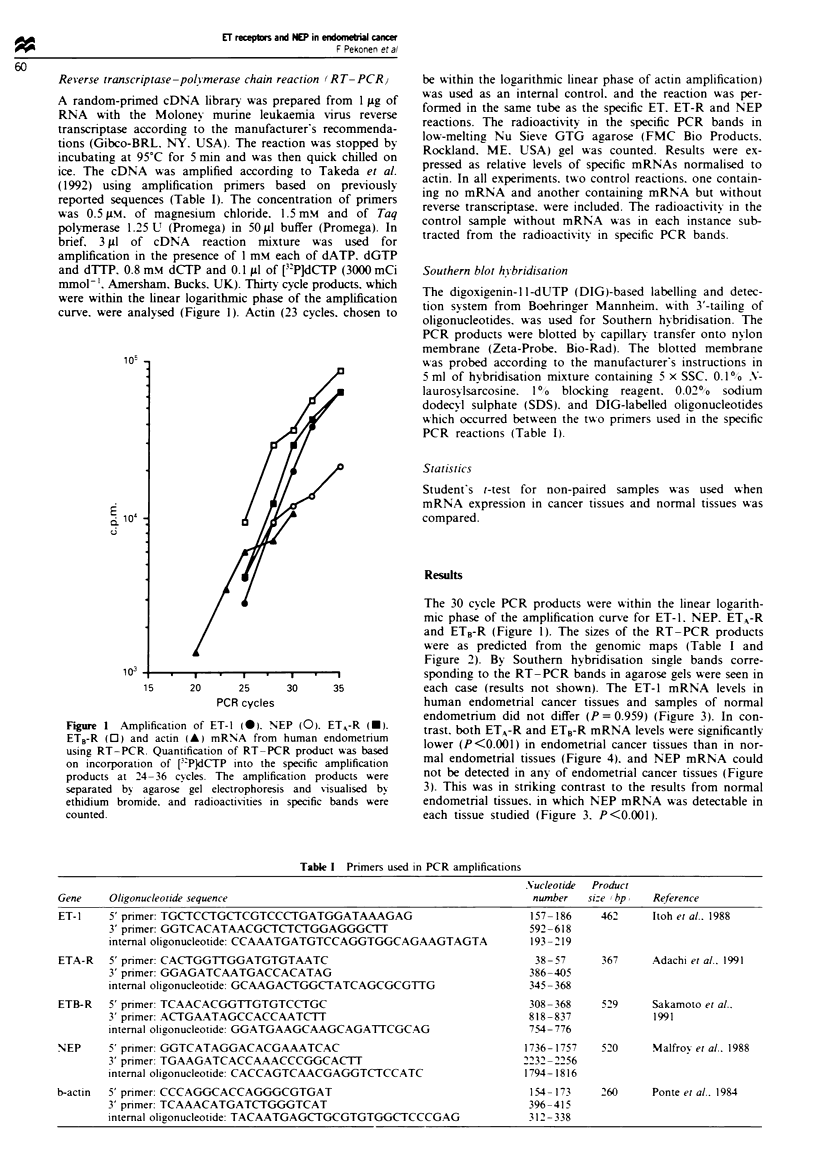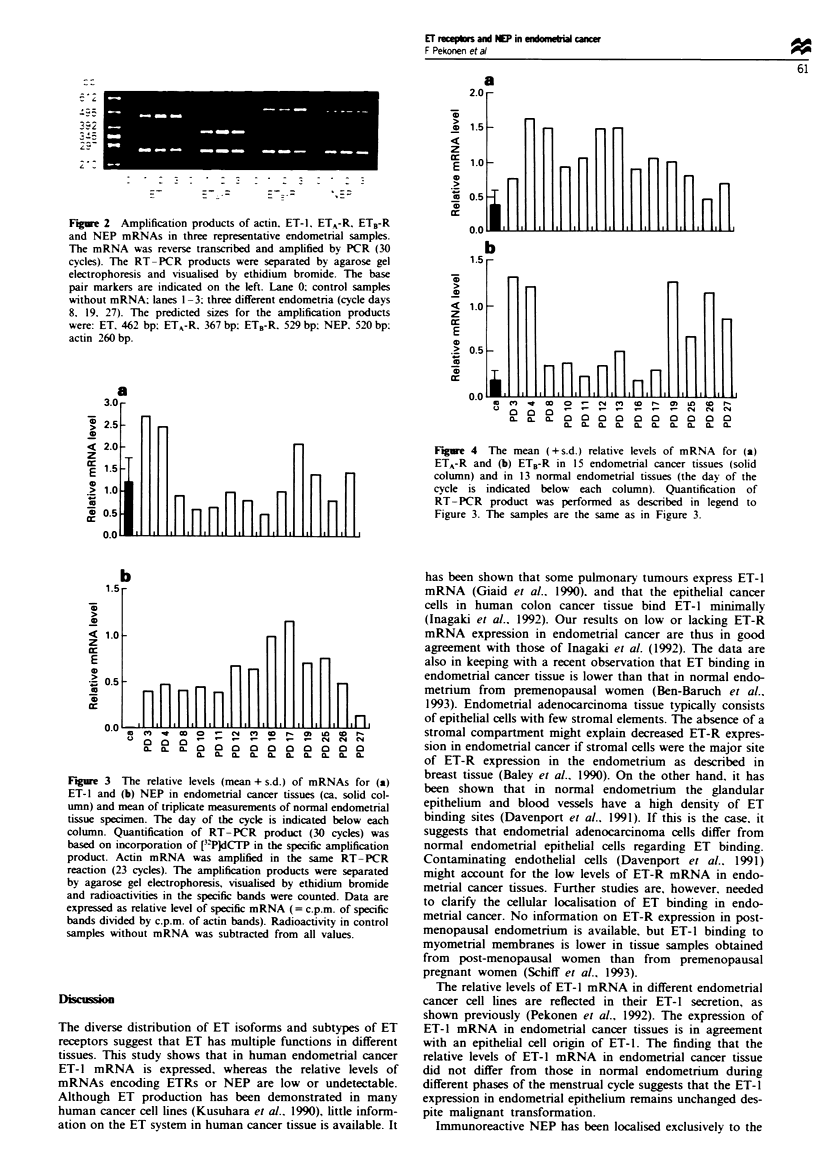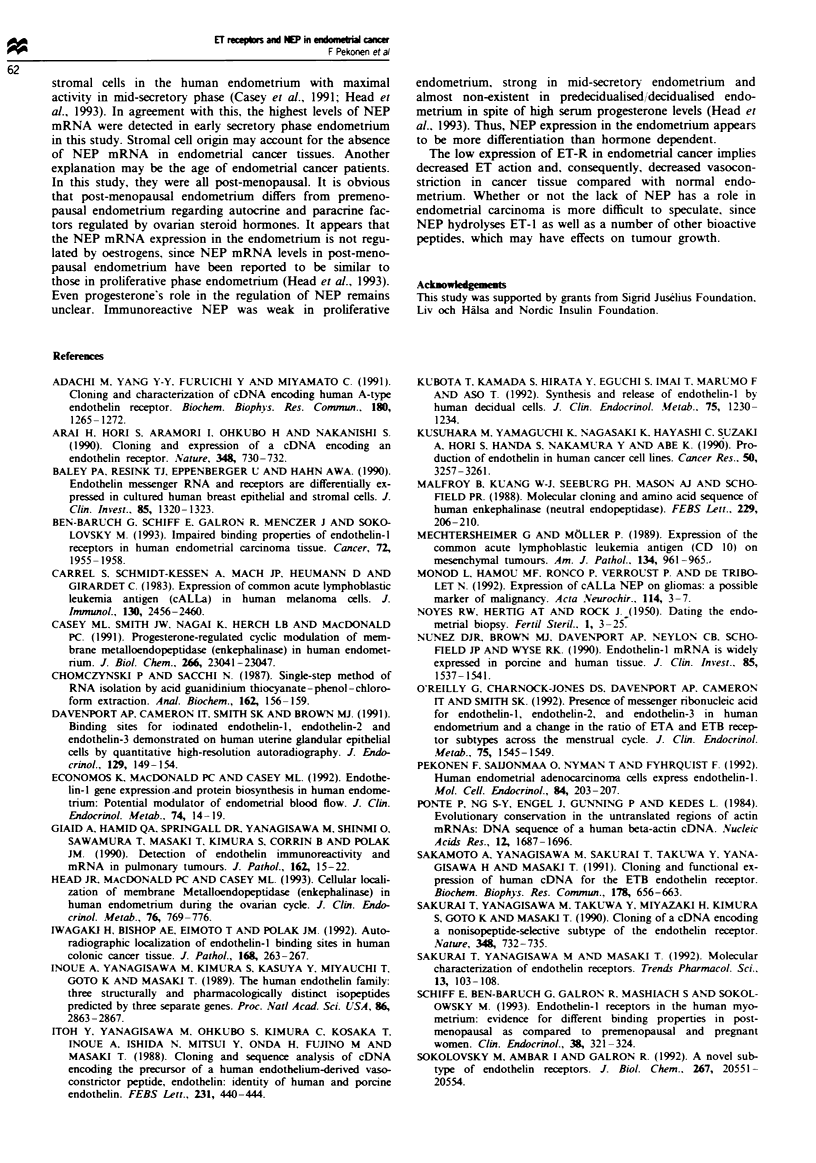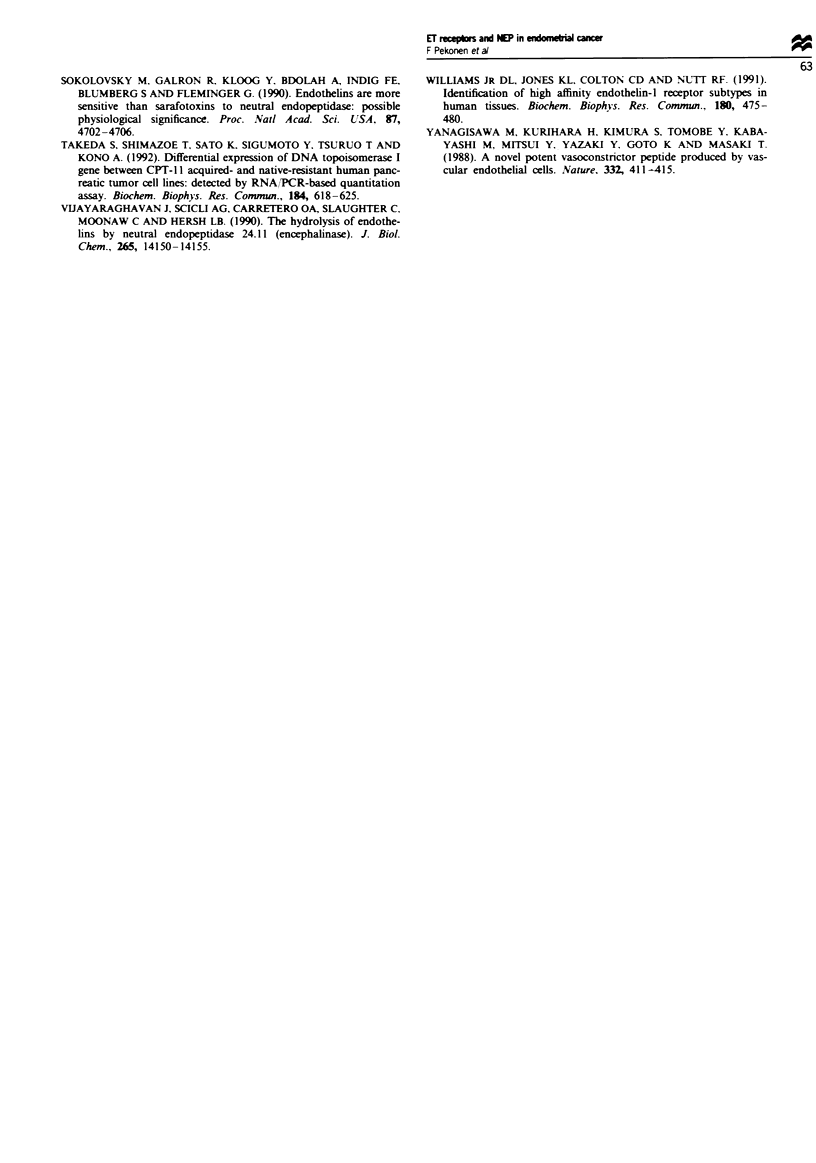# Decreased expression of messenger RNAs encoding endothelin receptors and neutral endopeptidase 24.11 in endometrial cancer.

**DOI:** 10.1038/bjc.1995.12

**Published:** 1995-01

**Authors:** F. Pekonen, T. Nyman, M. Ammälä, E. M. Rutanen

**Affiliations:** Minerva Institute for Medical Research, Helsinki.

## Abstract

**Images:**


© 1995 Stockton Press All rights reserved 0007–0920/95 $9.00

### Decreased expression of messenger RNAs encoding endothelin receptors and neutral endopeptidase 24.11 in endometrial cancer

F Pekonen[1], T Nyman[1], M Ämmälä[1] and E-M Rutanen[2]

[1]*Minerva Institute for Medical Research, Tukholmankatu 2, SF-00250 Helsinki;* [2]*Department II of Obstetrics and Gynecology, Helsinki University Central Hospital, SF-00290 Helsinki, Finland.*

**Summary** In this study, we used reverse transcriptase–polymerase chain reaction (RT–PCR) to compare the expression of mRNAs encoding endothelin-1 (ET-1), endothelin receptors type A ($ET_A$-R) and type B ($ET_B$-R) and ET-1-degrading enzyme neutral endopeptidase 24.11 (NEP) in 15 endometrial cancer tissues and 13 normal endometrial tissues. The relative levels of ET-1 mRNA in endometrial cancer tissues did not differ from those in normal endometrium. Both $ET_A$-R and $ET_B$-R mRNA levels were significantly lower in endometrial cancer tissue than in normal endometrium ($P<0.001$). The complete lack of NEP mRNA in endometrial cancer tissues was in marked contrast to results from normal endometrium ($P<0.001$). In conclusion, differential expression of mRNAs encoding ET-R and NEP in normal endometrium and endometrial cancer suggests that ET action is altered in endometrial cancer compared with normal endometrium.

**Keywords:** endothelin; receptor; endometrium; cancer; neutral endopeptidase

Endothelin (ET) is a 21 amino acid peptide which was first described in 1988 as a potent vasoconstrictor localised in vascular endothelium (Yanagisawa *et al.*, 1988). Subsequently, three ET isopeptides, called ET-1, ET-2 and ET-3, with different biological activities, have been identified by screening the human genomic DNA library (Inoue *et al.*, 1989). Although ET-1 was originally isolated from cultured endothelial cells, it has become evident that the ETs are widely distributed in different tissues and organs (Nunez *et al.*, 1990). The different potencies of the three isoforms of the ET family opened up the possibility of the existence of multiple ET receptor (ET-R) subtypes as well. Two distinct ET receptors with different specificities have been cloned (Arai *et al.*, 1990; Sakurai *et al.*, 1990). The type A endothelin receptor ($ET_A$-R) has high affinity for ET-1 and ET-2 but little cross-reactivity with ET-3 and sarafotoxin S6c, whereas type B receptor ($ET_B$-R) is non-selective with similar affinities for the different ETs and sarafotoxin S6c (Sakurai *et al.*, 1992). A third ET-R subtype with superhigh affinity has been described but not yet cloned (Sokolovsky *et al.*, 1992). The ET-Rs are widely distributed in cell lines and organs, but their relative abundance in different tissues varies. $ET_B$-R dominates in hippocampus, whereas $ET_A$-R dominates in the uterus (Williams *et al.*, 1991). Neutral endopeptidase 24.11 (NEP), also called enkephalinase, is a zinc-containing plasma membrane enzyme that efficiently degrades a number of small bioactive peptides, including the ETs (Sokolovsky *et al.*, 1990; Vijayaraghavan *et al.*, 1990).

In the human endometrium, ET-1 has been shown to be produced by both stromal and epithelial cells, with increased concentrations observed before and during menstruation (Economos *et al.*, 1992). The mRNAs for ET-1, ET-2 and ET-3 have been detected in human endometrium throughout the menstrual cycle (O'Reilly *et al.*, 1992). Also, pregnancy endometrium (decidua) synthesises ET-1 (Kubota *et al.*, 1992). The expression of $ET_A$-R and $ET_B$-R mRNA in the endometrium varies with the phase of the menstrual cycle, the ratio of $ET_A$-R to $ET_B$-R being lowest in late secretory phase (O'Reilly *et al.*, 1992). NEP is present in the human endometrium throughout the menstrual cycle (Head *et al.*, 1993). The specific activity of the enzyme is correlated with the plasma levels of progesterone and is highest in early and mid-secretory phase (Casey *et al.*, 1991).

Correspondence: F Pekonen
Received 20 April 1994; revised 26 August 1994; accepted 26 August 1994

Little information on the role of the ET system in cancer is available. Several cancer cell lines, including endometrial cancer cell lines, have been shown to produce ET (Kusuhara *et al.*, 1990; Pekonen *et al.*, 1992), but information on ET-R mRNA expression in cancer tissue is scarce. In colon cancer, decreased ET receptor activity has been reported (Inagaki *et al.*, 1992). Expression of NEP mRNA is typical for leukaemias, but has also been demonstrated in other malignancies such as melanomas, gliomas and mesenchymal tumours (Carrel *et al.*, 1983; Mechtersheimer and Möller, 1989; Monod *et al.*, 1992). Information on ET-1 mRNA expression in endometrial cancer tissue is lacking. Neither ET-R nor NEP mRNAs have been described in endometrial cancer tissue. In this study we compared the mRNA expression of ET-related substances in endometrial cancer and normal endometrium in an attempt to elucidate their potential role in the genesis of endometrial cancer.

### Materials and methods

### *Tissues*

Endometrial cancer tissue was obtained from 15 women who underwent hysterectomy for endometrial adenocarcinoma at the Department of Obstetric and Gynecology, Helsinki University Central Hospital. The mean age of the patients was 62.6 years (range 47–80 years). Samples of normal endometrium were obtained from 13 women who underwent laparoscopic tubal ligation at the Hyvinkää district Hospital, Finland. The mean age of these women was 42.2 years (range 35–47). The samples were collected with the approval of the Local Ethical Committees. The tissue samples were snap frozen in liquid nitrogen immediately after removal and stored at −80°C until processed. Routine haematoxylin and eosin-stained paraffin sections were prepared for histological evaluation. The dating of the endometrial samples was based on the first day of the last menstrual period and histological examination according to the method of Noyes *et al.* (1950).

### *RNA isolation*

All reagents used for RNA isolation were molecular biology reagents from Sigma (St Louis, MO, USA). The guanidium thiocyanate method (Chomczynski and Sacchi, 1987) was used to isolate total RNA.

*Reverse transcriptase–polymerase chain reaction (RT–PCR)*

A random-primed cDNA library was prepared from 1 μg of RNA with the Moloney murine leukaemia virus reverse transcriptase according to the manufacturer's recommendations (Gibco-BRL, NY, USA). The reaction was stopped by incubating at 95°C for 5 min and was then quick chilled on ice. The cDNA was amplified according to Takeda *et al.* (1992) using amplification primers based on previously reported sequences (Table I). The concentration of primers was 0.5 μM, of magnesium chloride, 1.5 mM and of *Taq* polymerase 1.25 U (Promega) in 50 μl buffer (Promega). In brief, 3 μl of cDNA reaction mixture was used for amplification in the presence of 1 mM each of dATP, dGTP and dTTP, 0.8 mM dCTP and 0.1 μl of [$^{32}$P]dCTP (3000 mCi $mmol^{-1}$, Amersham, Bucks, UK). Thirty cycle products, which were within the linear logarithmic phase of the amplification curve, were analysed (Figure 1). Actin (23 cycles, chosen to be within the logarithmic linear phase of actin amplification) was used as an internal control, and the reaction was performed in the same tube as the specific ET, ET-R and NEP reactions. The radioactivity in the specific PCR bands in low-melting Nu Sieve GTG agarose (FMC Bio Products, Rockland, ME, USA) gel was counted. Results were expressed as relative levels of specific mRNAs normalised to actin. In all experiments, two control reactions, one containing no mRNA and another containing mRNA but without reverse transcriptase, were included. The radioactivity in the control sample without mRNA was in each instance subtracted from the radioactivity in specific PCR bands.

**Figure 1** Amplification of ET-1 (●), NEP (○), $ET_A$-R (■), $ET_B$-R (□) and actin (▲) mRNA from human endometrium using RT–PCR. Quantification of RT–PCR product was based on incorporation of [$^{32}$P]dCTP into the specific amplification products at 24–36 cycles. The amplification products were separated by agarose gel electrophoresis and visualised by ethidium bromide, and radioactivities in specific bands were counted.

*Southern blot hybridisation*

The digoxigenin-11-dUTP (DIG)-based labelling and detection system from Boehringer Mannheim, with 3′-tailing of oligonucleotides, was used for Southern hybridisation. The PCR products were blotted by capillary transfer onto nylon membrane (Zeta-Probe, Bio-Rad). The blotted membrane was probed according to the manufacturer's instructions in 5 ml of hybridisation mixture containing 5 × SSC, 0.1% *N*-laurosylsarcosine, 1% blocking reagent, 0.02% sodium dodecyl sulphate (SDS), and DIG-labelled oligonucleotides which occurred between the two primers used in the specific PCR reactions (Table I).

*Statistics*

Student's *t*-test for non-paired samples was used when mRNA expression in cancer tissues and normal tissues was compared.

### Results

The 30 cycle PCR products were within the linear logarithmic phase of the amplification curve for ET-1, NEP, $ET_A$-R and $ET_B$-R (Figure 1). The sizes of the RT–PCR products were as predicted from the genomic maps (Table I and Figure 2). By Southern hybridisation single bands corresponding to the RT–PCR bands in agarose gels were seen in each case (results not shown). The ET-1 mRNA levels in human endometrial cancer tissues and samples of normal endometrium did not differ ($P = 0.959$) (Figure 3). In contrast, both $ET_A$-R and $ET_B$-R mRNA levels were significantly lower ($P<0.001$) in endometrial cancer tissues than in normal endometrial tissues (Figure 4), and NEP mRNA could not be detected in any of endometrial cancer tissues (Figure 3). This was in striking contrast to the results from normal endometrial tissues, in which NEP mRNA was detectable in each tissue studied (Figure 3, $P<0.001$).

**Table I** Primers used in PCR amplifications

| *Gene* | *Oligonucleotide sequence* | *Nucleotide number* | *Product size (bp)* | *Reference* |
|---|---|---|---|---|
| ET-1 | 5′ primer: TGCTCCTGCTCGTCCCTGATGGATAAAGAG | 157–186 | 462 | Itoh *et al.*, 1988 |
| | 3′ primer: GGTCACATAACGCTCTCTGGAGGGCTT | 592–618 | | |
| | internal oligonucleotide: CCAAATGATGTCCAGGTGGCAGAAGTAGTA | 193–219 | | |
| ETA-R | 5′ primer: CACTGGTTGGATGTGTAATC | 38–57 | 367 | Adachi *et al.*, 1991 |
| | 3′ primer: GGAGATCAATGACCACATAG | 386–405 | | |
| | internal oligonucleotide: GCAAGACTGGCTATCAGCGCGTTG | 345–368 | | |
| ETB-R | 5′ primer: TCAACACGGTTGTGTCCTGC | 308–368 | 529 | Sakamoto *et al.*, 1991 |
| | 3′ primer: ACTGAATAGCCACCAATCTT | 818–837 | | |
| | internal oligonucleotide: GGATGAAGCAAGCAGATTCGCAG | 754–776 | | |
| NEP | 5′ primer: GGTCATAGGACACGAAATCAC | 1736–1757 | 520 | Malfroy *et al.*, 1988 |
| | 3′ primer: TGAAGATCACCAAACCCGGCACTT | 2232–2256 | | |
| | internal oligonucleotide: CACCAGTCAACGAGGTCTCCATC | 1794–1816 | | |
| b-actin | 5′ primer: CCCAGGCACCAGGGCGTGAT | 154–173 | 260 | Ponte *et al.*, 1984 |
| | 3′ primer: TCAAACATGATCTGGGTCAT | 396–415 | | |
| | internal oligonucleotide: TACAATGAGCTGCGTGTGGCTCCCGAG | 312–338 | | |

**Figure 2** Amplification products of actin, ET-1, ET$_A$-R, ET$_B$-R and NEP mRNAs in three representative endometrial samples. The mRNA was reverse transcribed and amplified by PCR (30 cycles). The RT-PCR products were separated by agarose gel electrophoresis and visualised by ethidium bromide. The base pair markers are indicated on the left. Lane 0: control samples without mRNA; lanes 1-3: three different endometria (cycle days 8, 19, 27). The predicted sizes for the amplification products were: ET, 462 bp; ET$_A$-R, 367 bp; ET$_B$-R, 529 bp; NEP, 520 bp; actin 260 bp.

**Figure 3** The relative levels (mean + s.d.) of mRNAs for (**a**) ET-1 and (**b**) NEP in endometrial cancer tissues (ca, solid column) and mean of triplicate measurements of normal endometrial tissue specimen. The day of the cycle is indicated below each column. Quantification of RT-PCR product (30 cycles) was based on incorporation of [$^{32}$P]dCTP in the specific amplification product. Actin mRNA was amplified in the same RT-PCR reaction (23 cycles). The amplification products were separated by agarose gel electrophoresis, visualised by ethidium bromide and radioactivities in the specific bands were counted. Data are expressed as relative level of specific mRNA (= c.p.m. of specific bands divided by c.p.m. of actin bands). Radioactivity in control samples without mRNA was subtracted from all values.

**Figure 4** The mean (+s.d.) relative levels of mRNA for (**a**) ET$_A$-R and (**b**) ET$_B$-R in 15 endometrial cancer tissues (solid column) and in 13 normal endometrial tissues (the day of the cycle is indicated below each column). Quantification of RT-PCR product was performed as described in legend to Figure 3. The samples are the same as in Figure 3.

### Discussion

The diverse distribution of ET isoforms and subtypes of ET receptors suggest that ET has multiple functions in different tissues. This study shows that in human endometrial cancer ET-1 mRNA is expressed, whereas the relative levels of mRNAs encoding ETRs or NEP are low or undetectable. Although ET production has been demonstrated in many human cancer cell lines (Kusuhara *et al.*, 1990), little information on the ET system in human cancer tissue is available. It has been shown that some pulmonary tumours express ET-1 mRNA (Giaid *et al.*, 1990), and that the epithelial cancer cells in human colon cancer tissue bind ET-1 minimally (Inagaki *et al.*, 1992). Our results on low or lacking ET-R mRNA expression in endometrial cancer are thus in good agreement with those of Inagaki *et al.* (1992). The data are also in keeping with a recent observation that ET binding in endometrial cancer tissue is lower than that in normal endometrium from premenopausal women (Ben-Baruch *et al.*, 1993). Endometrial adenocarcinoma tissue typically consists of epithelial cells with few stromal elements. The absence of a stromal compartment might explain decreased ET-R expression in endometrial cancer if stromal cells were the major site of ET-R expression in the endometrium as described in breast tissue (Baley *et al.*, 1990). On the other hand, it has been shown that in normal endometrium the glandular epithelium and blood vessels have a high density of ET binding sites (Davenport *et al.*, 1991). If this is the case, it suggests that endometrial adenocarcinoma cells differ from normal endometrial epithelial cells regarding ET binding. Contaminating endothelial cells (Davenport *et al.*, 1991) might account for the low levels of ET-R mRNA in endometrial cancer tissues. Further studies are, however, needed to clarify the cellular localisation of ET binding in endometrial cancer. No information on ET-R expression in post-menopausal endometrium is available, but ET-1 binding to myometrial membranes is lower in tissue samples obtained from post-menopausal women than from premenopausal pregnant women (Schiff *et al.*, 1993).

The relative levels of ET-1 mRNA in different endometrial cancer cell lines are reflected in their ET-1 secretion, as shown previously (Pekonen *et al.*, 1992). The expression of ET-1 mRNA in endometrial cancer tissues is in agreement with an epithelial cell origin of ET-1. The finding that the relative levels of ET-1 mRNA in endometrial cancer tissue did not differ from those in normal endometrium during different phases of the menstrual cycle suggests that the ET-1 expression in endometrial epithelium remains unchanged despite malignant transformation.

Immunoreactive NEP has been localised exclusively to the stromal cells in the human endometrium with maximal activity in mid-secretory phase (Casey *et al*., 1991; Head *et al*., 1993). In agreement with this, the highest levels of NEP mRNA were detected in early secretory phase endometrium in this study. Stromal cell origin may account for the absence of NEP mRNA in endometrial cancer tissues. Another explanation may be the age of endometrial cancer patients. In this study, they were all post-menopausal. It is obvious that post-menopausal endometrium differs from premenopausal endometrium regarding autocrine and paracrine factors regulated by ovarian steroid hormones. It appears that the NEP mRNA expression in the endometrium is not regulated by oestrogens, since NEP mRNA levels in post-menopausal endometrium have been reported to be similar to those in proliferative phase endometrium (Head *et al*., 1993). Even progesterone's role in the regulation of NEP remains unclear. Immunoreactive NEP was weak in proliferative endometrium, strong in mid-secretory endometrium and almost non-existent in predecidualised/decidualised endometrium in spite of high serum progesterone levels (Head *et al*., 1993). Thus, NEP expression in the endometrium appears to be more differentiation than hormone dependent.

The low expression of ET-R in endometrial cancer implies decreased ET action and, consequently, decreased vasoconstriction in cancer tissue compared with normal endometrium. Whether or not the lack of NEP has a role in endometrial carcinoma is more difficult to speculate, since NEP hydrolyses ET-1 as well as a number of other bioactive peptides, which may have effects on tumour growth.

**Acknowledgements**

This study was supported by grants from Sigrid Jusélius Foundation, Liv och Hälsa and Nordic Insulin Foundation.

**References**

ADACHI M, YANG Y-Y, FURUICHI Y AND MIYAMATO C. (1991). Cloning and characterization of cDNA encoding human A-type endothelin receptor. *Biochem. Biophys. Res. Commun.*, **180**, 1265–1272.

ARAI H, HORI S, ARAMORI I, OHKUBO H AND NAKANISHI S. (1990). Cloning and expression of a cDNA encoding an endothelin receptor. *Nature*, **348**, 730–732.

BALEY PA, RESINK TJ, EPPENBERGER U AND HAHN AWA. (1990). Endothelin messenger RNA and receptors are differentially expressed in cultured human breast epithelial and stromal cells. *J. Clin. Invest.*, **85**, 1320–1323.

BEN-BARUCH G, SCHIFF E, GALRON R, MENCZER J AND SOKOLOVSKY M. (1993). Impaired binding properties of endothelin-1 receptors in human endometrial carcinoma tissue. *Cancer*, **72**, 1955–1958.

CARREL S, SCHMIDT-KESSEN A, MACH JP, HEUMANN D AND GIRARDET C. (1983). Expression of common acute lymphoblastic leukemia antigen (cALLa) in human melanoma cells. *J. Immunol.*, **130**, 2456–2460.

CASEY ML, SMITH JW, NAGAI K, HERCH LB AND MACDONALD PC. (1991). Progesterone-regulated cyclic modulation of membrane metalloendopeptidase (enkephalinase) in human endometrium. *J. Biol. Chem.*, **266**, 23041–23047.

CHOMCZYNSKI P AND SACCHI N. (1987). Single-step method of RNA isolation by acid guanidinium thiocyanate-phenol-chloroform extraction. *Anal. Biochem.*, **162**, 156–159.

DAVENPORT AP, CAMERON IT, SMITH SK AND BROWN MJ. (1991). Binding sites for iodinated endothelin-1, endothelin-2 and endothelin-3 demonstrated on human uterine glandular epithelial cells by quantitative high-resolution autoradiography. *J. Endocrinol.*, **129**, 149–154.

ECONOMOS K, MACDONALD PC AND CASEY ML. (1992). Endothelin-1 gene expression and protein biosynthesis in human endometrium: Potential modulator of endometrial blood flow. *J. Clin. Endocrinol. Metab.*, **74**, 14–19.

GIAID A, HAMID QA, SPRINGALL DR, YANAGISAWA M, SHINMI O, SAWAMURA T, MASAKI T, KIMURA S, CORRIN B AND POLAK JM. (1990). Detection of endothelin immunoreactivity and mRNA in pulmonary tumours. *J. Pathol.*, **162**, 15–22.

HEAD JR, MACDONALD PC AND CASEY ML. (1993). Cellular localization of membrane Metalloendopeptidase (enkephalinase) in human endometrium during the ovarian cycle. *J. Clin. Endocrinol. Metab.*, **76**, 769–776.

IWAGAKI H, BISHOP AE, EIMOTO T AND POLAK JM. (1992). Autoradiographic localization of endothelin-1 binding sites in human colonic cancer tissue. *J. Pathol.*, **168**, 263–267.

INOUE A, YANAGISAWA M, KIMURA S, KASUYA Y, MIYAUCHI T, GOTO K AND MASAKI T. (1989). The human endothelin family: three structurally and pharmacologically distinct isopeptides predicted by three separate genes. *Proc. Natl Acad. Sci. USA*, **86**, 2863–2867.

ITOH Y, YANAGISAWA M, OHKUBO S, KIMURA C, KOSAKA T, INOUE A, ISHIDA N, MITSUI Y, ONDA H, FUJINO M AND MASAKI T. (1988). Cloning and sequence analysis of cDNA encoding the precursor of a human endothelium-derived vasoconstrictor peptide, endothelin: identity of human and porcine endothelin. *FEBS Lett.*, **231**, 440–444.

KUBOTA T, KAMADA S, HIRATA Y, EGUCHI S, IMAI T, MARUMO F AND ASO T. (1992). Synthesis and release of endothelin-1 by human decidual cells. *J. Clin. Endocrinol. Metab.*, **75**, 1230–1234.

KUSUHARA M, YAMAGUCHI K, NAGASAKI K, HAYASHI C, SUZAKI A, HORI S, HANDA S, NAKAMURA Y AND ABE K. (1990). Production of endothelin in human cancer cell lines. *Cancer Res.*, **50**, 3257–3261.

MALFROY B, KUANG W-J, SEEBURG PH, MASON AJ AND SCHOFIELD PR. (1988). Molecular cloning and amino acid sequence of human enkephalinase (neutral endopeptidase). *FEBS Lett.*, **229**, 206–210.

MECHTERSHEIMER G AND MÖLLER P. (1989). Expression of the common acute lymphoblastic leukemia antigen (CD 10) on mesenchymal tumours. *Am. J. Pathol.*, **134**, 961–965.

MONOD L, HAMOU MF, RONCO P, VERROUST P AND DE TRIBOLET N. (1992). Expression of cALLa NEP on gliomas: a possible marker of malignancy. *Acta Neurochir.*, **114**, 3–7.

NOYES RW, HERTIG AT AND ROCK J. (1950). Dating the endometrial biopsy. *Fertil Steril.*, **1**, 3–25.

NUNEZ DJR, BROWN MJ, DAVENPORT AP, NEYLON CB, SCHOFIELD JP AND WYSE RK. (1990). Endothelin-1 mRNA is widely expressed in porcine and human tissue. *J. Clin. Invest.*, **85**, 1537–1541.

O'REILLY G, CHARNOCK-JONES DS, DAVENPORT AP, CAMERON IT AND SMITH SK. (1992). Presence of messenger ribonucleic acid for endothelin-1, endothelin-2, and endothelin-3 in human endometrium and a change in the ratio of ETA and ETB receptor subtypes across the menstrual cycle. *J. Clin. Endocrinol. Metab.*, **75**, 1545–1549.

PEKONEN F, SAIJONMAA O, NYMAN T AND FYHRQUIST F. (1992). Human endometrial adenocarcinoma cells express endothelin-1. *Mol. Cell. Endocrinol.*, **84**, 203–207.

PONTE P, NG S-Y, ENGEL J, GUNNING P AND KEDES L. (1984). Evolutionary conservation in the untranslated regions of actin mRNAs: DNA sequence of a human beta-actin cDNA. *Nucleic Acids Res.*, **12**, 1687–1696.

SAKAMOTO A, YANAGISAWA M, SAKURAI T, TAKUWA Y, YANAGISAWA H AND MASAKI T. (1991). Cloning and functional expression of human cDNA for the ETB endothelin receptor. *Biochem. Biophys. Res. Commun.*, **178**, 656–663.

SAKURAI T, YANAGISAWA M, TAKUWA Y, MIYAZAKI H, KIMURA S, GOTO K AND MASAKI T. (1990). Cloning of a cDNA encoding a nonisopeptide-selective subtype of the endothelin receptor. *Nature*, **348**, 732–735.

SAKURAI T, YANAGISAWA M AND MASAKI T. (1992). Molecular characterization of endothelin receptors. *Trends Pharmacol. Sci.*, **13**, 103–108.

SCHIFF E, BEN-BARUCH G, GALRON R, MASHIACH S AND SOKOLOWSKY M. (1993). Endothelin-1 receptors in the human myometrium: evidence for different binding properties in post-menopausal as compared to premenopausal and pregnant women. *Clin. Endocrinol.*, **38**, 321–324.

SOKOLOVSKY M, AMBAR I AND GALRON R. (1992). A novel subtype of endothelin receptors. *J. Biol. Chem.*, **267**, 20551–20554.

SOKOLOVSKY M, GALRON R, KLOOG Y, BDOLAH A, INDIG FE, BLUMBERG S AND FLEMINGER G. (1990). Endothelins are more sensitive than sarafotoxins to neutral endopeptidase: possible physiological significance. *Proc. Natl Acad. Sci. USA*, **87**, 4702–4706.

TAKEDA S, SHIMAZOE T, SATO K, SIGUMOTO Y, TSURUO T AND KONO A. (1992). Differential expression of DNA topoisomerase I gene between CPT-11 acquired- and native-resistant human pancreatic tumor cell lines: detected by RNA/PCR-based quantitation assay. *Biochem. Biophys. Res. Commun.*, **184**, 618–625.

VIJAYARAGHAVAN J, SCICLI AG, CARRETERO OA, SLAUGHTER C, MOONAW C AND HERSH LB. (1990). The hydrolysis of endothelins by neutral endopeptidase 24.11 (encephalinase). *J. Biol. Chem.*, **265**, 14150–14155.

WILLIAMS JR DL, JONES KL, COLTON CD AND NUTT RF. (1991). Identification of high affinity endothelin-1 receptor subtypes in human tissues. *Biochem. Biophys. Res. Commun.*, **180**, 475–480.

YANAGISAWA M, KURIHARA H, KIMURA S, TOMOBE Y, KABAYASHI M, MITSUI Y, YAZAKI Y, GOTO K AND MASAKI T. (1988). A novel potent vasoconstrictor peptide produced by vascular endothelial cells. *Nature*, **332**, 411–415.